# When cholesterol is not cholesterol: a note on the enzymatic determination of its concentration in model systems containing vegetable extracts

**DOI:** 10.1186/1476-511X-9-65

**Published:** 2010-06-21

**Authors:** Mariona Jové, José CE Serrano, Maria Josep Bellmunt, Anna Cassanyé, Neus Anglès, Jordi Reguant, José R Morelló, Reinald Pamplona, Manuel Portero-Otín

**Affiliations:** 1 Institut de Recerca Biomèdica de Lleida-Universitat de Lleida-Parc Científic i Agroalimentari Tecnològic de Lleida (IRBLLEIDA-UdL-PCiTAL). c/Montserrat Roig, 25008 Lleida, Spain; 2 La Morella Nuts, SA. Apel.les Mestres, S/N 43006 Reus, Spain

## Abstract

**Background:**

Experimental evidences demonstrate that vegetable derived extracts inhibit cholesterol absorption in the gastrointestinal tract. To further explore the mechanisms behind, we modeled duodenal contents with several vegetable extracts.

**Results:**

By employing a widely used cholesterol quantification method based on a cholesterol oxidase-peroxidase coupled reaction we analyzed the effects on cholesterol partition. Evidenced interferences were analyzed by studying specific and unspecific inhibitors of cholesterol oxidase-peroxidase coupled reaction. Cholesterol was also quantified by LC/MS. We found a significant interference of diverse (cocoa and tea-derived) extracts over this method. The interference was strongly dependent on model matrix: while as in phosphate buffered saline, the development of unspecific fluorescence was inhibitable by catalase (but not by heat denaturation), suggesting vegetable extract derived H_2_O_2 _production, in bile-containing model systems, this interference also comprised cholesterol-oxidase inhibition. Several strategies, such as cholesterol standard addition and use of suitable blanks containing vegetable extracts were tested. When those failed, the use of a mass-spectrometry based chromatographic assay allowed quantification of cholesterol in models of duodenal contents in the presence of vegetable extracts.

**Conclusions:**

We propose that the use of cholesterol-oxidase and/or peroxidase based systems for cholesterol analyses in foodstuffs should be accurately monitored, as important interferences in all the components of the enzymatic chain were evident. The use of adequate controls, standard addition and finally, chromatographic analyses solve these issues.

## Background

Cholesterol function is essential for membrane physiology, bile acids and steroid hormones biosynthesis. However, an elevated level of cholesterol in plasma is implicated in atherosclerosis and other cardiovascular diseases [1,2]. Therefore, minimizing dietary cholesterol intake is often recommended as a primary measure for lowering cholesterolemia [3]. In the intestinal tract, dietary lipids are first emulsified in the lumen by bile components (biliary salts and phospholipids) and then encapsulated into micelles. Cholesterol can be then transferred from micelles to gut wall and thereafter to bloodstream [4]. Epidemiological and experimental evidence demonstrate that the consume of vegetable foods allows to a lowering effect on cholesterol plasma levels and diminished risk of atherosclerosis progression [5,6]. It is known that cholesterol esters, phenol compounds and other vegetable derived nutrients can block the entry of most cholesterol into micelles, partially preventing its absorption [7]. While developing a model of "in vitro" digestion based on published methods [8] we analyzed mixtures of cholesterol with selected foodstuffs and bile, we detected strong interferences in a widely used method for cholesterol quantification arising from different vegetable foods like cocoa and/or green tea.

This study characterized those potential interferences and presents different solutions to solve them.

## Results

### Vegetable extracts induce an apparent increase in cholesterol content in "in vitro" experiments using an enzymatic method

**Figure 1:**
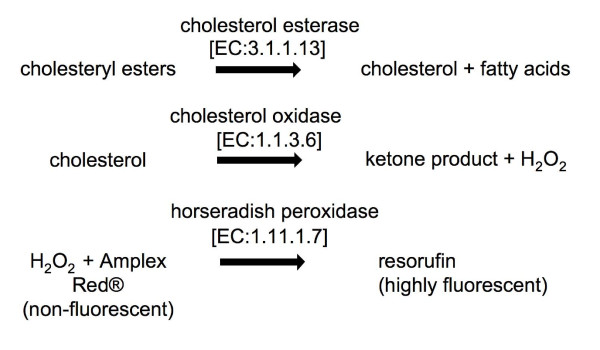
**Enzymatic method used for quantification of cholesterol based on cholesterol oxidase-peroxidase coupled reaction**. Amplex Red^®^: 10-acetyl-3, 7-dihidroxyphenoxazine.

**Figure 2:**
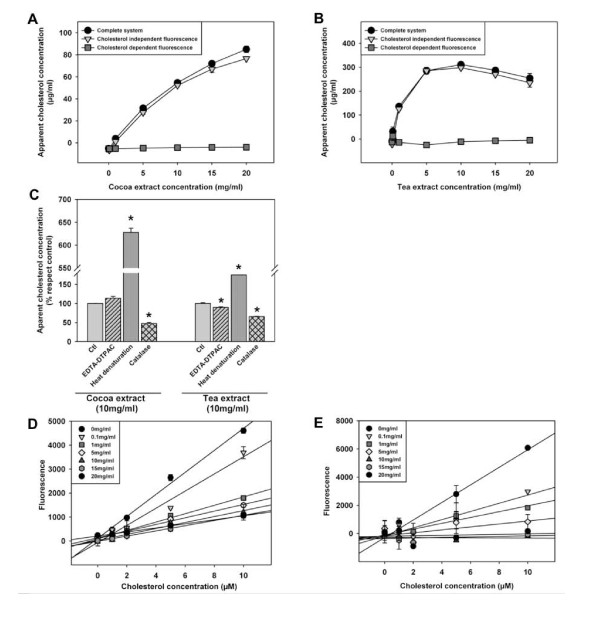
**Vegetable extracts interfere with cholesterol analyses in phosphate buffered saline based systems using cholesterol oxidase peroxidase-coupled reactions**. Both cocoa (a) and tea-derived (b) extracts showed, in a dose dependent fashion, reactivity in systems used for cholesterol analyses using cholesterol oxidase-peroxidase coupled reactions. Cholesterol independent fluorescence was defined as fluorescence arising from the complete system without the enzymes cholesterol oxidase and cholesterol esterase. Cholesterol content was obtained by subtracting cholesterol independent fluorescence from the complete system. c. Interference was not sensible to heat-denaturation (96°C, 3 min) or metal chelation (EDTA and DTPAC 1 mM), but to catalase (7 mg/ml). Linearity of a cholesterol standard curve was sensible to the presence of either cocoa (d) and tea-derived (e) extracts diluted in phosphate buffered saline in different concentrations (from 0 to 20 mg/ml). Values are means ± SEM. Statistical analysis was done by ANOVA followed by Tukey HSD post hoc test (* p < 0.05).

The presence of vegetable derived extracts in a duodenal micelle model gave rise to its apparent cholesterol content using a commercial method based on a cholesterol-oxidase coupled reaction (Figure 1). This kit is also recommended by the manufacturer for the use in food extracts. Different vegetable extracts (in concentration ranging 0 to 20 mg/ml) were analyzed for the potential interference in the cholesterol assay. Cocoa and tea extracts increased the apparent cholesterol concentration (figure 2A and 2B) in a concentration dependent fashion, even in the absence of cholesterol oxidase, the key enzyme in this system. Considering the enzyme-coupled reaction of this method (Figure 1), we analyzed (in comparison to the complete system), i) the cholesterol independent fluorescence (system without cholesterol esterase and cholesterol oxidase), ii) the peroxidase activity independent fluorescence and iii) the resorufin independent fluorescence. Fluorescence found in both cholesterol dependent and independent conditions suggested either a generation of vegetable extract derived H_2_O_2 _and/or an interference from peroxidase activity present in vegetable extracts.

Phytoesterol interferences were ruled out as ergosterol and other sterols (data not shown) did not offer fluorescence in the complete system. Metal chelation (EDTA-DTPAC) did not inhibit significantly fluorescence in the complete system in the presence of vegetable extracts except in the case of tea extract (figure 2C). The potential contribution of vegetable derived peroxidases was ruled out by heat denaturation of vegetable extracts, even resulting in the increase of fluorescence (figure 2C). Finally, the inhibitory activity of catalase [1.11.1.6] (leading to fluorescence decreases to 50%) suggests that there is a vegetable extract dependent H_2_O_2 _production which may be derived by the previously described interaction of sample antioxidants with horseradish peroxidase [9]. Moreover, in the presence of vegetable extracts, the fluorescence in both cholesterol-dependent and independent reactions offered similar values suggesting the possibility of using the cholesterol independent fluorescence as a blank to account the vegetable extract derived interferences (figures 2A and 2B).

To test this alternative solution, cholesterol standard curves in the presence of vegetable derived extracts were developed (figure 2D and 2E). The slope of the cholesterol concentration-fluorescence linear relationship was inversely related to the concentration of vegetable extract, in a given range (up to 10 mg/ml in cocoa extract). Therefore, it is advisable to develop a cholesterol standard curve with the chosen concentration of vegetable extract in order to ascertain linearity, even in the presence of the cholesterol independent fluorescence blank. It should be noted that in specific cases the interference of the vegetable extracts precluded the use of cholesterol standard curve even at a lower doses (from 1 mg/ml in tea).

### Vegetable extracts induce an apparent decrease in cholesterol incorporation in bile micelles. Chromatographic approach for cholesterol analysis

**Figure 3:**
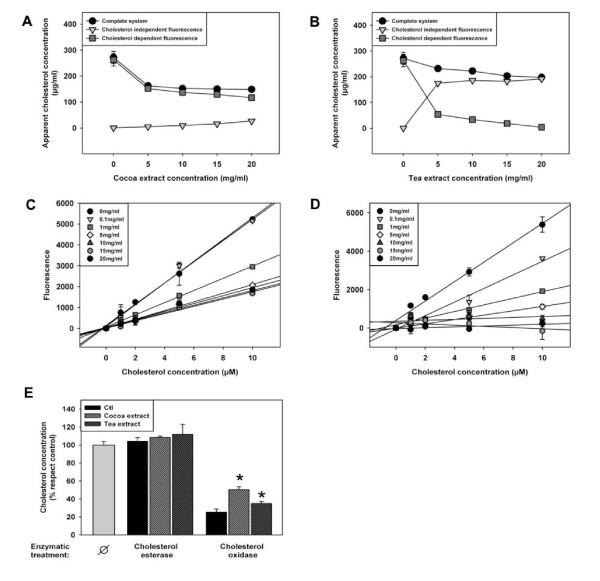
**Vegetable extracts interfere with cholesterol analyses in in the presence of porcine bile based systems using cholesterol oxidase peroxidase-coupled reactions**. Both cocoa (a) and tea-derived (b) extracts inhibited, in a dose dependent fashion, bile-derived cholesterol reactivity in systems used for cholesterol analyses using cholesterol oxidase-peroxidase coupled reactions. Cholesterol-dependent and independent fluorescences were as defined in figure 2. Linearity of a cholesterol standard curve was sensible to the presence of either cocoa (c) and tea-derived (d) extracts diluted in bile in different concentrations (from 0 to 20 mg/ml). (e) Vegetable-extracts (10 mg/mL) inhibited cholesterol oxidase activity in the presence of bile, based on the cholesterol chromatographic assay. Values are means ± SEM. Statistical analysis was done by ANOVA followed by Tukey HSD post hoc test (* p < 0.05).

In order to further model duodenal conditions, the extracts were diluted with porcine bile as previously described [8]. The fluorescence was analyzed in cholesterol dependent and independent conditions. Surprisingly, the cholesterol independent fluorescence (i.e. vegetable extract derived H_2_O_2_) disappeared (cocoa extract) or decreased (tea extract) in this condition (figure 3A and 3B). Nevertheless, a novel interference was detected, since apparent cholesterol concentration decreased. In order to further characterize this new interference, a standard curve using different extract concentration was done. The results (figure 3C and 3D) show the same behavior of cholesterol standard curves as in the case of PBS, i.e., the slope of the cholesterol standard curve was reduced when the concentration of food extract was increased.

In order to further characterize those interferences in duodenal-like conditions, we used a chromatographic approach. Figure 3E shows that the cholesterol concentration does not change with the addition of cholesterol esterase, probably due to the virtual absence of cholesteryl esters in porcine bile. The addition of cholesterol oxidase in the mixture resulted in a strong decrease in the cholesterol detection because most of the cholesterol was oxidized (Figure 3E). However, the addition of vegetable derived extracts caused an increase in cholesterol content in those conditions, suggesting an inhibitory activity of those extracts over cholesterol oxidase in the presence of bile.

**Figure 4:**
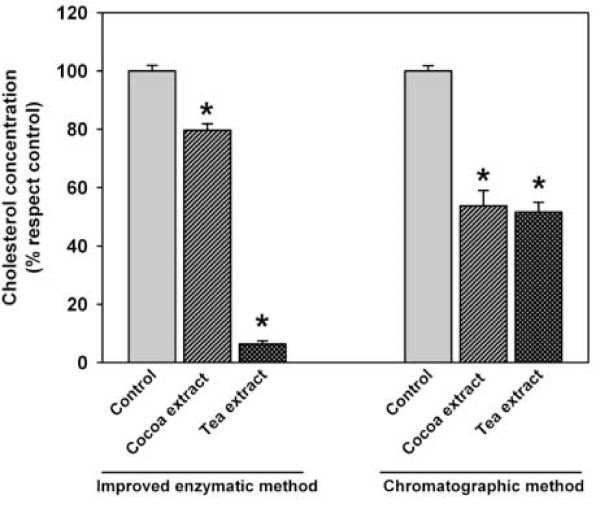
**Vegetable extract impairs cholesterol bioavailability in a model of duodenal content, but this impairment could be overestimated (tea) or underestimated (cocoa) by enzyme-based cholesterol assays**. Values are means ± SEM. Statistical analysis was done by ANOVA followed by Tukey HSD post hoc test (* p < 0.05).

To further elucidate whether vegetable extracts induced a decrease in the cholesterol in the micellar (bioavailable) phase, cocoa and tea extracts were diluted in bile, incubated, centrifuged and filtered to obtain the micellar fraction as previously described [8]. Next, the cholesterol content in this fraction was analyzed both by the enzyme-based assay and by the chromatographic method. The results show that, while porcine bile-vegetable interferences impeded accurate quantification by the enzyme-based assay, chromatographic method allowed it (Figure 4). The results also show that while enzyme-based assay showed marked differences in the potential for diminishing cholesterol absorption between both vegetable extracts, the use of chromatographic assays disclosed similar effects. With these results we concluded that although cholesterol-esterase and cholesterol-oxidase based assays are widely [10-12] used to measure cholesterol, it is necessary to develop artifact controls in order to offer an accurate measurement in the presence of foodstuffs.

## Discussion

There are several methods described to measure cholesterol content in foodstuff using chromatographic approaches [13,14]. However, when it is necessary to analyze a high number of samples, an enzymatic method based on the cholesterol oxidase-peroxidase coupled reaction could be the best option [10-12] if we take into account some interferences reported here.

With the objective of modeling the capability of vegetable extracts decreasing the bioavailable micellar cholesterol, we found different interferences in the measurement of cholesterol using an enzymatic method. In phosphate buffered saline, we evidenced a highly intense cholesterol independent fluorescence that we could attribute partially (50%) to an exogenous H_2_O_2 _production, as in those conditions it was diminished by catalase preincubation. The rest of the interference could be explained by a different mechanism recently described in our group [9], showing an interaction of vegetable antioxidants with peroxidases. Horseradish peroxidase is often used as final step for enzymatic-coupled reactions. Briefly, in the absence of hydrogen peroxide but in aerobic conditions, the antioxidant compound of the vegetable extracts could reduce the ferric-horseradish peroxidase to ferrous-horseradish peroxidase and then interact with the O_2 _of the medium producing horseradish peroxidase-Compound III. This compound can undergo spontaneous decay to ferriperoxidase with the generation of O_2_^- ^which may interact with the antioxidant producing an antioxidant radical. This antioxidant radical may then react with the Amplex Red^® ^and horseradish peroxidase and produce resorufin. Thus, all peroxidase based enzymatic methods may show interferences by those vegetable derived compounds. To solve these interferences we propose two different and complementary methods: i) the use of cholesterol independent condition as a blank of the reaction to eliminate the fluorescence when the vegetable extracts are diluted in PBS and ii) the application of cholesterol standard curves including the working concentration of the extract.

For the studies of "in vitro" cholesterol absorption we used porcine bile in order to reproduce a physiologically relevant and a stable source of micellar cholesterol. We first analyze the interference of the extracts diluted in bile and we found a novel interference that decreases the apparent cholesterol concentration. In this case, the use of the cholesterol independent fluorescence is not as useful as in the case of buffered saline-based systems because in this latter case the interference is lower. However, the cholesterol and/or cholesterol ester standard curve addition with the extracts could be a good method to reduce the interferences detecting cholesterol at low concentrations of vegetable extracts. After incubating the extracts in the presence or in the absence of the enzymes cholesterol esterase and cholesterol oxidase we discover that vegetable extracts can interact directly and/or indirectly decrease the activity of cholesterol oxidase. We cannot exclude that other vegetable derived extracts could inhibits cholesterol esterase. The mechanisms behind this inhibitory effect are outside the scope of this work, but they may comprise displacement of cholesterol oxidase outside of lipid bilayers needed for efficient catalytic turnover [15] and they may be part of the defensive properties of polyphenols presents in vegetable extracts [16]. In any case, cholesterol oxidase is among the more frequently used enzymes and its interference should be accounted when extending the use from clinical chemistry to food chemistry [17].

Finally, the development of a chromatographic method in order to measure the cholesterol concentration in biological models of food digestion is presented as a solution when the used concentration of foodstuff is high enough to interfere with the standard addition method. Both detection methods were used to test the effect of these extracts in lowering the concentration of potentially bioavailable cholesterol. Moreover, it is described that sterol, polyphenols and other nutrients can block the entry of most cholesterol into micelles [8,18-20]. Although there were evidenced a blocking effect of cholesterol entry into micellar phases by both extracts using both methods the magnitude of the change differed. Using the enzymatic method we demostrated that the cholesterol independent fluorescence in the case of tea extract was very high, quite similar to the fluorescence of complete system. This high fluorescence resulted in a small detection of real cholesterol dependent fluorescence. In the case of cocoa extract the decrease in apparent bioavailability is about 20%. Analyzing the cholesterol concentration using a chromatographic method both extracts at tested concentrations decreased micellar cholesterol about a 50%.

## Conclusions

The use of cholesterol-oxidase and/or peroxidase based systems for cholesterol analyses in foodstuffs should be accurately monitored, as important interferences in all the components of the enzymatic chain were evident. The use of adequate controls, standard addition and finally, chromatographic analyses should solve these issues.

## Methods

### Chemicals

Ethylenediaminetetraacetic acid (EDTA), diethylenetriaminepentaacetic acid (DTPAC), ergosterol, catalase, chloroform and cholesterol were from Sigma (Sigma-Aldrich, Saint Louis, MO, USA). Acetonitrile, 2-propanol, ammonium acetate and formic acid were from Baker (Mallinckrodt Baker, Phillipsburg, NJ, USA). Millex GP filters 0,22 μm and Ultrafree-MC filtres 30,000 from Millipore (Millipore, Billerica, MA, USA), methanol from Carlo Erba (Carlo Erba, Milano, Italy) and [25,26,26,26,26,27,27,27-^2^H_7_]cholesterol (cholesterol-D7) from Avanti Polar Lipids (Avanti Polar Lipids Inc, Alabaster, AL, USA).

Bile from porcine biliary vesicle was collected from a local abattoir and placed on ice. Immediately after collection, bile was centrifuged at 2000 g at 4°C for 10 min to remove debris. The cholesterol content of the bile was measured (see below) and then the bile was diluted with phosphate buffered saline to obtain a 0.5 mM of cholesterol concentration, as described by Kirana et al [8]. It was aliquoted and stored at -80°C.

Green tea and cocoa vegetable extracts were provided by La Morella Nuts SA (La Morella Nuts, Reus, Spain).

### Cholesterol measurement using an enzymatic method

The concentration of cholesterol in porcine bile, food extracts and mixes was analyzed by *Amplex Red Cholesterol Assay Kit (A12216) *based on an enzymatic reaction depicted in figure 1. Briefly, reactions took place in a 96 well plate by the addition of 50 μL of Amplex Red working solution with 50 μL of assay sample. Five mL of working solution, prepared prior the analysis, contained 75 μL of a 300 μM of Amplex Red reagent and 2 U/mL of HRP. The working solution volume was adjusted to 5 mL with reaction buffer, which contained 25 mM potassium phosphate, pH 7.4, 12.5 mM NaCl, 1.25 mM cholic acid and 0.025% Triton X-100. The reactions were incubated for 30 min at 37°C, protected from light. After incubation, fluorescence was measured in a fluorescence microplate reader (Tecan Infinite M200, Männedorf, Switzerland) using excitation wavelength at 560 nm and emission detection at 590 nm.

The samples were suspended with either phosphate buffered saline or porcine bile to obtain the initial desired concentration of nutrient (from 0 to 20 mg/ml). After a dilution in PBS (1:25) cholesterol amount was quantified by fluorescence according the kit instructions.

To model the cholesterol entry into micellar phases and its potential inhibition by vegetable extracts, the method of Kirana et al was used [8]. Briefly, samples suspended in pig's bile at 10 mg/ml were incubated at 37°C for 1 h with continuous shaking at 160 r.p.m. (Unitron, Infors HT, Headquarter, Switzerland). The solution was then centrifuged at 1000 × g for 10 min, filtered through a 0.22 μm Millex GP and diluted 25 fold. The concentration of cholesterol was analyzed by *Amplex Red Cholesterol Assay Kit (A12216)*.

### Cholesterol analysis by liquid chromatography coupled to mass-spectrometry

To unequivocally quantify cholesterol in food extract-bile mixtures, cholesterol was extracted using chloroform:methanol (2:1) as described previously [21,22]. Previous to extraction cholesterol-D7 was added, as an internal standard, to a final concentration of 125 μg/mL. The mixture was vortexed and centrifuged at 4400 × g at room temperature for 15 min. The organic phase was conserved and the procedure was repeated. Combined organic phases were dried in a SpeedVac (Thermo Fisher Scientific, Madrid, Spain) and dissolved with methanol. The samples were filtered in an UltraFree 5 kDa filter (Millipore, Billerica, MA, USA) before liquid chromatography analysis.

Liquid chromatography was done in an Agilent LC model G2226A coupled to an ESI-QTOF MS 6520 (Agilent Technologies, Barcelona, Spain). For this purpose, extracted sample was applied onto a reverse-phase column (C18 Luna 3 micron pfp(2) 100 A 150 × 2 mm, Phenomenex, California, USA), equipped with a guard-column kept at 50°C. The flow rate was 100 μl/min with solvent A composed of water containing 1% ammonium acetate 1 M, 0.1% formic acid, and solvent B composed of acetonitrile:2-propanol (5:2; v/v) containing 1% ammonium acetate 1 M, 0.1% formic acid. The gradient started from 65% A/35% B, reached 100% B in 35 min, held there for the next 10 min and re-equilibrated for 15 min [23]. The temperature of the sample organizer was set at 10°C.

Data were collected in positive electrospray mode TOF operated in full-scan mode at 100 to 3000 m/z. The capillary voltage was 3500 V with a scan rate of 1 scan/s. N_2 _was used as a gas nebulizer (Flow: 5 L/min and T = 350°C). The Masshunter Software was used for integration and extraction of peak intensities of the cholesterol and cholesterol-D7. The *m/z *values used for quantification were: *m/z *369.35 [M+H-H2O]^+ ^for cholesterol and *m/z *376.3955 [M+H-H2O]^+ ^for cholesterol-D7. Interassay and intraassay variation < 5%, L.O.Q was 20 pmol, RSQ = 0.98.

## Competing interests

The authors declare that they have no competing interests.

## Authors' contributions

MJ and JCES performed analytical determinations and designed part of the research. MJB and RP performed statistical analyses. NA, JR and JRM obtained the extract of interest. MJ, NA and MPO conceived the study and participated in its design and coordination and helped to draft the manuscript. All authors read and approved the final manuscript.
